# Using a daily diary for monitoring intrusive memories of trauma: A translational data synthesis study exploring convergent validity

**DOI:** 10.1002/mpr.1936

**Published:** 2022-08-17

**Authors:** Laura Singh, Sara Ahmed Pihlgren, Emily A. Holmes, Michelle L. Moulds

**Affiliations:** ^1^ Department of Psychology Uppsala University Uppsala Sweden; ^2^ Swedish Collegium for Advanced Study Uppsala Sweden; ^3^ Department of Clinical Neuroscience Karolinska Institutet Stockholm Sweden; ^4^ School of Psychology UNSW Sydney Sydney New South Wales Australia

**Keywords:** diary, intrusive memories, psychological trauma, psychometrics, validation study

## Abstract

**Objective:**

Intrusive memories are a core feature of posttraumatic stress disorder and have transdiagnostic relevance across mental disorders. Establishing flexible methods to monitor intrusions, including patterns and characteristics, is a key challenge. A daily diary has been developed in experimental settings to provide symptom count data, without the need for retrospective self‐report over extended time periods (e.g., 1 week, 1 month). We conducted an exploratory, pre‐registered data synthesis investigating convergence between the diary and questionnaire measures of intrusive symptoms long used in clinical practice (Impact of Event Scale, IES, and revised version, IES‐R, Intrusion subscale).

**Results:**

Utilising datasets using the daily diary from 11 studies (4 real‐world trauma studies, seven analogue trauma studies; total *N* = 578), we found significant positive associations between the diary and IES/IES‐R Intrusion subscale. Exploratory analyses indicated that the magnitude of this association was stronger for the IES (vs. the IES‐R), and in individuals with real‐world (vs. analogue) trauma.

**Conclusion:**

This study provides first evidence of convergent validity of a daily diary for monitoring intrusions with a widely used questionnaire. A diary may be a more flexible methodology to obtain information about intrusions (frequency, characteristics, triggers, content), relative to questionnaires which rely on retrospective reporting of symptoms over extended timeframes. We discuss potential benefits of daily monitoring of intrusions in clinical and research contexts.

## 1 INTRODUCTION

Mental health researchers and clinicians employ a range of approaches to index psychiatric phenomena in the laboratory and the clinic. Most common among these are validated self‐report questionnaires with established psychometric properties. A plethora of such measures now exist; they index an array of variables including but not limited to mood, cognition, behaviour (e.g. avoidance; Cloitre et al., 2018; Hyland et al., 2017) and levels of psychopathology (e.g. anxiety and depression; Zigmond & Snaith, 1983). Instruments have been developed to measure both state and trait variables; for example, to index respondents' mood at the time of completion (‘right now’), as opposed to a more general or trait‐like way of responding (e.g., ‘typically’, ‘over the last month’).

Focussing on specific target symptoms rather than full, multifaceted psychiatric diagnoses (Fernandes et al., 2017; Parker et al., 2017) might help offer new ways of thinking in mental health science and the potential for deriving new treatment approaches (Holmes et al., 2014; Singh et al., 2020). Intrusive memories are mental imagery‐based impressions of a traumatic event that repeatedly intrude into the mind unwanted (Iyadurai et al., 2019). They can elicit significant distress and are a core clinical feature of both acute stress disorder and posttraumatic stress disorder (American Psychiatric Association, 2013; Singh et al., 2020), but also experienced in other disorders such as depression, social anxiety and panic disorder (Hackmann & Holmes, 2004; Holmes et al., 2021; Newby & Moulds, 2011).

Intrusive memories are often assessed with validated self‐report questionnaires (Frans et al., 2005). The Intrusion subscale of the Impact of Event Scale (IES; Horowitz et al., 1979) and the revised version, the Impact of Event Scale‐Revised (IES‐R; Weiss & Marmar, 1997) provides a self‐report measure of intrusive symptoms that has been widely used within both research and clinical practice given that it has been freely available since the 1980s. While it is not the clinical gold standard for assessing intrusive symptoms such as the Clinician‐Administered PTSD Scale (CAPS, a clinician administered structured interview which requires at least two intrusive memories over the past month for a PTSD diagnosis; Weathers et al., 2018), it has pragmatic value and has helped shape much research in the field of trauma. This includes both clinical research with patients after real‐world trauma and experimental psychopathology research with non‐clinical participants exposed to an experimental analogue of a traumatic event.

The IES (Horowitz et al., 1979) asks respondents to rate how frequently they experienced intrusive symptoms (e.g., intrusive memories) of a traumatic event during the past week, while the IES‐R (Weiss & Marmar, 1997) indexes the extent to which intrusive symptoms are experienced as distressing, also anchored to the past week. Whilst such measures enable researchers to measure changes in psychological processes, a key shortcoming is that they commonly rely on retrospective recall over long periods—thus factors such as memory biases and mood state may influence responding. Further, respondents categorise how often they experience intrusive symptoms (IES) or the degree of distress associated with these symptoms (IES‐R), using verbal descriptors such as ‘often’ (IES) or ‘extremely’ (IES‐R). Accordingly, responses are essentially reliant on estimates. This approach is all the more problematic when researchers are interested in more precise patterns of symptoms across a given period (Santangelo et al., 2018), particularly of intermittent and spontaneous phenomena such as intrusive memories. That is, an individual's estimates of their ‘average’ number of intrusions provides no information about daily or weekly fluctuations. Such information may highlight triggers and clustering of intrusions, and would be clinically informative. In light of these challenges, alternative methodologies such as daily diaries in which participants are instructed to monitor their intrusions as (or close to the time) they occur (Holmes et al., 2004, 2009, 2010), in real‐time (Palmier‐Claus et al., 2011), have been developed (see Bolger et al., 2003; Bolger & Laurenceau, 2013; Iida et al., 2012 for a review of the applications of diary methods; see also Rattel et al., 2019).

Whilst daily diaries are informative and flexible, one limit to current knowledge is the absence of research examining whether the number of memories recorded in such diaries corresponds with responses on validated self‐report measures. A number of studies (Holmes et al., 2009; Iyadurai et al., 2018; James et al., 2015; Kanstrup et al., 2021; Singh et al., 2021) have shown consistent downstream effects of experimental manipulations on intrusive memories, both when intrusions are monitored in a daily diary and indexed by the Intrusion subscale of both the IES and IES‐R—providing preliminary evidence of correspondence. However, to our knowledge, no study to date has validated the daily diary employed in the intrusive memory literature against such questionnaires.

### 1.1 Aims of the study

Our aim was to explore the degree to which the number of intrusions reported in a daily diary are associated with a widely used questionnaire which indexes intrusive re‐experiencing for which psychometric properties have been established. We utilized data from both laboratory (i.e., non‐clinical) studies and clinical studies in which participants: (i) used a daily diary to record the number of intrusive memories of an analogue trauma (i.e., film clips of traumatic events) or a real‐life traumatic event they experienced over the past week,^1^ and (ii) completed the intrusion subscale of either the IES or IES‐R anchored to the corresponding timeframe.

We tested the hypothesis that the number of intrusions reported in the daily diary would be significantly correlated with Intrusion subscale scores on the IES and IES‐R. We collapsed experimental conditions investigated in the original studies in this analysis, reasoning that these two indices of intrusion content should be associated irrespective of any experimental manipulation.

## 2 METHOD

### 2.1 Participants

Five hundred and seventy eight participants were included in the dataset, which was comprised of data from four studies with clinical samples and seven laboratory‐based experimental studies with non‐clinical participants (see Table 1). Participants (*N* = 578; 41% female^2^) were aged from 18 to 65 years (*M* = 24.69, *SD* = 6.81^3^), and the majority were recruited from the general community.

**TABLE 1:** List of datasets included in the pooled data analyses and participant/study characteristics

Experiment	OSF	*N*	Participant characteristics					Intrusive memory diary characteristics		IES/IES‐R
			Age, *M (SD)*	Gender (*n*)		Location	Sample	Diary duration	Diary accuracy, *M (SD)*	
				Female	Male					
Laboratory										
Holmes et al. (2009)	N/A	40	23.05 (5.42)	18	22	UK	Non‐clinical	7 days	7.70 (2.60)^a^	IES
Lang et al. (2009)	N/A	48	29.52 (10.89)	24	24	UK	Non‐clinical	7 days	8.42 (1.07)^b^	IES
James et al. (2015)—EXP 1	https://osf.io/ideta/	52	24.46 (6.05)	31	21	UK	Non‐clinical	7 days	8.27 (1.52)^b^	IES‐R
James et al. (2015)—EXP 2	https://osf.io/ideta/	72	25.72 (8.51)	47	25	UK	Non‐clinical	7 days	8.40 (1.21)^b^	IES‐R
Woud, Blackwell, et al. (2018)	N/A	94	23.09 (3.64)	72	22	Germany	Non‐clinical	7 days	8.33 (1.56)^b^	IES‐R
Woud, Cwik, et al. (2018)	N/A	36^c^	23.19 (4.37)	17	19	UK	Non‐clinical	24 h	N/A	IES‐R
Porcheret et al. (2019)	N/A	50	24.20 (4.16)	27	21^d^	UK	Non‐clinical	6 days	N/A	IES‐R
Clinical										
Iyadurai et al. (2018)	https://osf.io/e4hc7/	67^e^	N/A	N/A	N/A	UK	Patients from ED	7 days	8.25 (1.62)^a^	IES‐R
Porcheret et al. (2020)	https://osf.io/fsn2f/	84^f^	N/A	N/A	N/A	UK	Patients from ED^g^	7 days	7.94 (1.45)^a^	IES‐R
Kanstrup et al. (2021)	https://osf.io/nma5q/	32^h^	N/A	N/A	N/A	Sweden	Patients from ED	7 days	8.55 (1.45)^a^	IES‐R
Singh et al. (2021)	All data reported within the manuscript	3	∼50	3	0	Sweden	Nurses who experienced work‐related trauma	7 days	8.00 (3.46)^a^	IES‐R

Abbreviations: ED, Emergency Department; IES, Impact of Event Scale; IES‐R, Impact of Event Scale‐Revised; M, Mean; OSF, Open Science Framework; SD, standard deviation.

^a^ Accuracy rating was an 11‐point scale (0–10).

^b^ Accuracy rating was a 10‐point scale (1–10).

^c^ The original paper had 38 participants, however due to missing diary data from one participant and missing IES‐R data from another participant, only *n* = 36 were available for analysis.

^d^ One participant had missing age/gender data and one participant indicated they prefer not to answer the gender question.

^e^ Only per‐protocol participants.

^f^ Three participants had missing diary data, therefore only 84 participants were included in analysis.

^g^ Had experienced/witnessed a traumatic event.

^h^ Only per‐protocol participants, *n* = 30 rated diary accuracy and an additional seven participants had missing data for IES‐R and could not be included in analysis.

Inclusion criteria for the current study were as follows: studies (i) authored by the Research Guarantor (Prof. Emily Holmes) between 2009 and July 2021,^4^ (ii) in which the number of intrusive memories was assessed with a daily diary, and the IES/IES‐R Intrusion subscale was administered, and (iii) for which we had access to the primary data (i.e., the number of intrusive memories reported in the diary and the IES/IES‐R Intrusion subscale scores).^5^ These inclusion criteria resulted in 11 datasets from 10 published studies at the time the current study was preregistered (see https://osf.io/au6d4/, published November 22, 2021). Using the per‐protocol data from these studies resulted in a dataset consisting of 578^6^ participants (*n* for clinical studies = 186, *n* for laboratory studies = 392).

### 2.2 Outcome measures

#### 2.2.1 Intrusive memory diary (Holmes et al., 2004, 2009, 2010)

Participants monitored the occurrence of intrusive memories (of the trauma film clips in laboratory studies, of a real‐life traumatic event in clinical studies) in a pen and paper diary (or digital adaptation thereof; Singh et al., 2021), recording the number of memories they experienced each day. Whilst some adaptations were made to the instructions and some features of the diary across studies (e.g., reporting the total of number of memories per day vs. reporting memories across four daily timepoints—morning, afternoon, evening, night), the core structure of the diary was consistent across all of the studies included. All research staff were trained in how to deliver the diary. The outcome variable was the frequency of intrusive memories, here operationalized as the mean number of intrusions per day (i.e., mean number of intrusions per day, over a diary period of x days). That is, whilst in the majority of studies included, intrusion frequency was reported as a 1‐week total, some employed shorter diary durations (range between 24 h and 8 days) (Kanstrup, Kontio, et al., 2021; Woud, Blackwell, et al., 2018). For the purpose of the current analysis across studies, we thus operationalize intrusion frequency as mean number of intrusive memories per day.

#### 2.2.2 Diary accuracy rating

In some studies, a bespoke rating scale was used to assess participants' self‐rated accuracy of their completion of the intrusive memory diary (rated on the last day the diary; see Table 1 for details). In most included studies, accuracy was assessed on a 10‐point scale (from 1 = *not at all accurate* to 10 = *extremely accurate*), although in some studies a 11‐point scale was used (from 0 = *not at all* to 10 = *extremely*), with minor variations in wording of question/answering options (Iyadurai et al., 2018; Kanstrup et al., 2021).

#### 2.2.3 Impact of Event Scale (IES; Horowitz et al., 1979) and Impact of Event Scale—Revised (IES‐R; Weiss & Marmar, 1997)

The IES is a 15‐item self‐report measure of traumatic stress symptoms with two subscales: intrusion and avoidance. Participants rate the frequency of each symptom over the past 7 days on a 4‐point scale, from 0 = *not at all* to 5 = *often*. The intrusion subscale of the IES possesses good reliability (*α* = 0.86) (Sundin & Horowitz, 2002).

The revised version (IES‐R) contains 22 items with three subscales: intrusion, avoidance and hyperarousal. Some items of the original IES were slightly modified in the IES‐R (e.g., the IES uses ‘I had trouble falling asleep or staying asleep because of pictures or thoughts about it that came into my mind’, while the IES‐R modified the item to ‘I had trouble falling asleep’), and the rating scale was altered to a 5‐point scale from 0 = *not at all* to 4 = *extremely*. In addition, the instructions were altered such that respondents rate the *distress* caused by each symptom during the past 7 days, rather than its *frequency*. The IES‐R possesses excellent internal consistency (*α* = 0.96) and converges with other measures of posttraumatic stress symptomology, for example, the PTSD checklist (*r* = 0.84) (Creamer et al., 2003).

Example items included in both the IES and IES‐R are follows: ‘Pictures about it popped into my mind’ and “I thought about it when I didn't mean to” (referring to the traumatic event).

### 2.3 Procedure

Data were included from 11 studies (four clinical, seven non‐clinical) from 10 publications (see Table 1). All participants provided their written and informed consent.

Participants in all studies were given both verbal and written instructions about how to complete the intrusive memory diary. These instructions included a definition of intrusive memories (i.e., *mental images from the film clips or traumatic event that come to mind spontaneously*) to ensure that monitoring was limited to this specific type of intrusion; that is, did not include other cognitions associated with the trauma film clips or traumatic event (e.g., rumination, verbal thoughts, voluntary memories).

Participants were instructed to use the diary to record any intrusive memories they experienced over a specified period (in most studies 7 days) and to record ‘*0*’ if they did not experience any intrusive memories during this period. Participants were instructed to carry the diary with them throughout the week, and to record any intrusive memories as they occurred; that is, not just complete the monitoring once within a specified time period (e.g., in the evening). Participants in the clinical studies monitored intrusive memories of their index traumatic event (e.g. a car crash, an assault); in the laboratory studies, participants viewed a trauma film (James et al., 2016) made up of a series of clips depicting traumatic scenes (e.g., of a car accident) and monitored intrusions of the film. To verify that intrusions reported in the laboratory studies were in fact of footage from the trauma film, participants were asked to include a brief description of the content of each intrusion in the diary. To increase the likelihood of compliance and accuracy of monitoring, participants were asked to carry the diary with them at all times. In addition, in some studies participants were sent reminders to complete the diary in order to maximise compliance (James et al., 2015; Kanstrup et al., 2021).

Participants returned the intrusive memory diary and completed the IES or IES‐R (Intrusion subscale) with responses anchored to the past week,^7^ either during a follow‐up appointment (James et al., 2015) or via post/online (Kanstrup et al., 2021).

### 2.4 Statistical analyses

#### 2.4.1 Data validation checks

To ensure we had the correct raw data from each study, we first confirmed whether we could reproduce the key statistics for the outcome variables (number of intrusions in daily diary and IES/IES‐R intrusion subscale score) as reported in the original articles. Validation was possible for all but one dataset (Iyadurai et al., 2018) (see Table 1), in which random sampling procedures imputed missing data for four participants. For the present study, which for practical purposes takes a per‐protocol rather than intention‐to‐treat approach, we excluded these four participants (we note that their data are available in the open‐access provided data). Additionally, we also performed validation checks for age and reproduced the statistics reported for these variables in the original articles.

#### 2.4.2 Pre‐registered analysis

To examine the association between the number of intrusive memories (mean per day) recorded in the daily diary and participants' IES/IES‐R Intrusion subscale score, a Spearman's rank‐order correlational analysis was conducted (given that we hypothesized a monotonic but not necessarily linear relation between the two outcome variables). Since the IES and IES‐R used different answering scales, standardized scores were used for data analysis. To further explore the association between the two variables visually, a scatterplot is presented for visual inspection.

#### 2.4.3 Additional exploratory (i.e., post hoc) analyses

Correlational analyses (Spearman's rank) were conducted separately between the number of intrusive memories (mean per day) and participants' IES Intrusion subscale score and the IES‐R Intrusion subscale score, given the different instructions used in the two versions of the questionnaire (i.e., *frequency* of intrusive symptoms in the IES vs. *distress* related to intrusive symptoms in the IES‐R). Finally, following initial visual inspection of the results, exploratory correlational analyses were conducted between the number of intrusive memories (mean per day) recorded in the daily diary and participants' (standardized) IES/IES‐R Intrusion subscale score separately for laboratory and clinical studies.

## 3 RESULTS

The mean number of intrusive memories reported per day in the daily diary across all participants was 1.10 (*SD* = 1.96, range 0–17, *n* = 578). Mean score on the IES was 7.88 (*SD* = 5.60, range 0–23, *n* = 88); mean score on the IES‐R was 5.62 (*SD* = 6.46, range 0–28, *n* = 490). The distribution of intrusion diary data as well as IES and IES‐R scores was positively skewed.

### 3.1 Association between the number of intrusive memories in the diary and the IES/IES‐R Intrusion subscales combined

**FIGURE 1:**
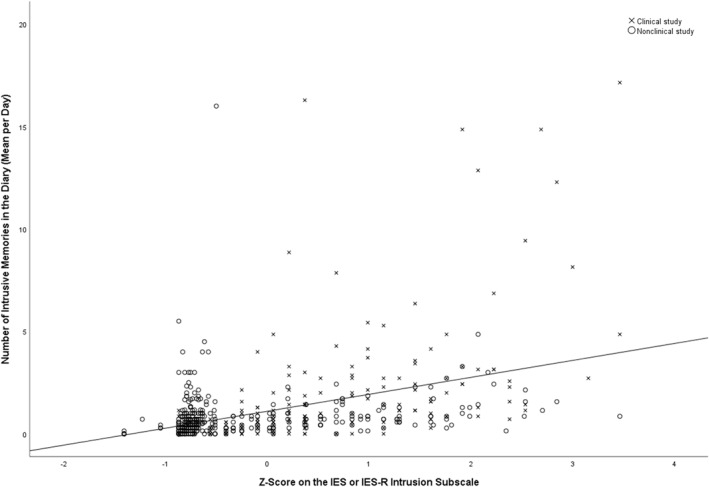
Scatterplot presenting the association between the number of intrusive memories in the diary (mean per day) and standardized scores on the IES/IES‐R Intrusion subscale

There was a moderate (Cohen, 1988; Cohen et al., 2003), positive correlation between the number of intrusive memories (mean per day) recorded in the daily diary and participants' standardized IES/IES‐R Intrusion subscale score (*r*_*s*_(576) = 0.487, *p* < 0.001, two‐sided test). A scatterplot showing the association between the two variables for visual inspection is presented in Figure 1.

### 3.2 Associations between the number of intrusive memories in the diary and the Intrusion subscale of the IES and IES‐R separately

Given that the instructions of the IES ask respondents to rate the frequency of intrusive symptoms (as opposed to the IES‐R, which instructs respondents to rate the degree to which intrusive symptoms are distressing), it is more closely aligned to the intrusive memory diary. We therefore explored the association between the number of intrusive memories in the daily diary and the Intrusion subscale of the IES and IES‐R separately. Within the two studies (*n* = 88) using the IES, there was a strong (Cohen, 1988), positive correlation between the number of intrusive memories (mean per day) recorded in the daily diary and participants' Intrusion subscale scores (*r*_*s*_(86) = 0.586, *p* < 0.001, two‐sided test). Within the nine studies (*n* = 490) using the IES‐R, there was a moderate (Cohen, 1988), positive correlation between the number of intrusive memories (mean per day) in the diary and Intrusion subscale scores (*r*_*s*_(488) = 0.481, *p* < 0.001, two‐sided test).

### 3.3 Associations between the number of intrusive memories in the diary and the Intrusions subscale for laboratory and clinical studies separately

For the clinical studies (*n* = 186), there was a strong (Cohen, 1988), positive correlation between the number of intrusive memories (mean per day) recorded in the daily diary and participants' standardized IES/IES‐R Intrusion subscale score (*r*_*s*_(184) = 0.696, *p* < 0.001, two‐sided test). For the non‐clinical studies (*n* = 392), there was a moderate (Cohen, 1988), positive correlation between the number of intrusive memories (mean per day) recorded in the daily diary and participants' standardized IES/IES‐R Intrusion subscale score (*r*_*s*_(390) = 0.382, *p* < 0.001, two‐sided test).

## 4 DISCUSSION

We sought to validate the daily diary employed to monitor intrusive memories in a growing body of trauma research by addressing a simple but important question: do the number of intrusive memories monitored in a daily diary significantly correspond with a widely used questionnaire measure of intrusions with established psychometric properties? Across a series of clinical and laboratory studies using the daily diary, we found significant positive relationships with the Intrusion subscale of the IES and the IES‐R. Whilst this relationship was significant in clinical studies (of individuals following real‐life trauma) and laboratory studies (of individuals who viewed a trauma film), interestingly, exploratory analyses indicated a stronger association in the former. Additional exploratory analyses of the original and revised versions of the IES yielded strong and moderate correlations between the diary and the IES and IES‐R Intrusion subscale, respectively. That the relationship between the number of intrusive memories reported in the diary and the original IES was stronger in magnitude likely reflects the fact that the IES instructs respondents to rate intrusion frequency; that is, the instructions of the two instruments are more closely aligned. Nonetheless, the IES‐R (an index of intrusion‐related distress) was significantly correlated with intrusions, confirming convergence of the diary and both versions of this measure.

Our findings have implications for the assessment of intrusive memories in both research and practice. Given the strong convergence between the diary and a well‐established measure of intrusive symptoms, they raise the possibility that using a simple daily diary to monitor intrusive memories in real life may be just as informative in understanding the occurrence of intrusions as asking participants to complete a questionnaire. Moreover, the diary has the additional advantage of shedding light on specific details that cannot be captured via a measure which asks for a retrospective estimate of the frequency or impact of intrusions over the past week; for example, the time of day that intrusions are most likely to occur, patterns/clusters/fluctuations across the day, and intrusion triggers. Such rich information is potentially informative for both researchers and practitioners. Furthermore, the diary requires minimal input from a participant and is thus easy to complete; indeed, recently developed digital versions of the diary (Singh et al., 2021, 2022) further simplify its implementation. Another benefit of a simple symptom count in a diary (as compared to a questionnaire score) is that the data, as well as any meaningful change, are easy for both patients and clinicians to interpret, providing instant feedback. For these reasons, the daily diary may prove to be a welcome alternative to administering a lengthy battery of self‐report measures that will reduce client burden in both clinical and research settings and provide a rich array of data.

Having established that the daily diary and IES/IES‐R are convergent indices of intrusions, our findings prompt further questions regarding the validation of the diary that await future research. First, is there an association between the number of intrusions reported in the daily diary and indices of functioning (e.g., interpersonal, occupational) as established in other mental disorders (Faurholt‐Jepsen et al., 2019)? Future studies in this line of work in which researchers systematically assess functioning across multiple domains will determine whether such a relationship exists (Singh et al., 2022). Second, what reduction in intrusive memories reported in the daily diary might be considered clinically significant? That is, what difference (i.e., reduction) in the number of intrusive memories reported from pre‐ to post‐intervention equates to clinically meaningful change? Randomised controlled trials evaluating interventions for intrusive memories which include both diagnostic instruments/interviews and the daily diary as outcome measures are needed to answer these questions (Singh et al., 2022).

A key limitation of our data synthesis was that all of the datasets included were drawn from studies carried out by our research group. The rationale for analysing only our own research groups' data rather than conducting a meta‐study (Baribault et al., 2018) was foremost practical: participant‐level data from our studies were readily available and easily validated. However, as a consequence, we recognize that any conclusions that can be drawn are limited to our own data. Replication studies including datasets from a range of research groups and conducted with a range of participant samples are needed to confirm whether the relationships we found between the daily diary and IES also emerge more broadly (Varma et al., 2021). We note that the database we created for the purpose of the current study may be extended in the future in order to include data from other research groups; this will facilitate examining whether the evidence of convergent validity reported here is replicated. A second limitation is that although participants were instructed to monitor their intrusions in real‐time (i.e., as they occurred), we cannot rule out the possibility that some may have recorded intrusions at the end of a specified period (e.g., at the end of the morning, evening), or at the end of each day; that is, after a brief delay. Nonetheless, given that any such potential delay was likely brief (e.g., within a few hours), we see great advantage in using a daily diary to capture intrusions over questionnaires which draw on retrospective estimates over longer timeframes (e.g., 1 week, 1 month). That said, we acknowledge that event‐based ESM or random time‐based ESM approaches have the potential to circumvent this limitation and yield rich and informative data on intrusions (e.g., see Malik et al., 2014; Rattel et al., 2019). Related to this, in the future more studies are needed to investigate the potential advantages of digital diaries over pen and paper formats (e.g., utilizing our recently developed digital versions of the diary; Singh et al., 2021, 2022). Finally, although the diary is time efficient for respondents (relative to completing an extensive battery of questionnaires), we cannot rule out the possibility that daily monitoring of intrusions and receiving reminders to fill in the diary may increase some individuals' awareness of their frequency or provoke intrusions, which may in turn elicit distress. Such effects of the diary and the impact of reminders should be investigated in future research. We also acknowledge that since the diary measure and the questionnaires did converge, for some research questions it might in fact be easier and more practical to use retrospective reporting on an established questionnaire rather than the diary.

In sum, in a series of laboratory and clinical studies using a daily diary to monitor intrusive memories, we found significant positive relationships with the Intrusion subscale of both the IES and the IES‐R. Exploratory analyses showed that the magnitude of these associations were stronger when study participants: (i) had experienced a real‐life traumatic event, as opposed to when they viewed a trauma film (i.e., an analogue trauma), and (ii) completed the original IES, rather than the IES‐R. The correspondence between the daily diary and a reliable, established and well‐used questionnaire such as the IES/IES‐R raises the possibility that utilising a diary to monitor intrusions may be preferable to administering an extensive battery of measures since it more flexibly offers insights of value to both researchers and practitioners, and is a readily understandable means by which patients can track their symptoms. Future studies which include datasets from other research groups need to test whether our findings replicate. Establishing the link between the number of intrusive memories recorded in the diary and functional impairment, and determining the reduction in intrusions reported in the diary which equates to clinically meaningful change both represent two important future directions.

## AUTHOR CONTRIBUTIONS

Conceptualization (Laura Singh, Emily A. Holmes, Michelle L. Moulds), Methodology (all), Validation (Laura Singh, Sara Ahmed Pihlgren), Formal analysis, data curation, visualization (Laura Singh), Writing – Original Draft (Laura Singh, Michelle L. Moulds), Supervision (Emily A. Holmes, Michelle L. Moulds), Funding acquisition (Emily A. Holmes). All authors critically revised the manuscript approved the final version for publication.

## CONFLICT OF INTEREST

EAH reports serving on the board of trustees of the charity MQ: Transforming Mental Health but receives no remuneration for this role. EAH receives royalties from books and occasional fees for workshops and invited addresses; she receives occasional consultancy fees from the Swedish agency for health technology assessment and assessment of social services. The other authors report no competing interests.

## Data Availability

De‐identified summary data for the majority of datasets included in this synthesis are available on the Open Science Framework (see Table 1 for details). Data from the remaining studies supporting the current findings are available from the corresponding author upon reasonable request.

## ENDNOTES

^1^
Some studies used a shorter timeframe.

^2^
Percentage based on *N* = 394 participants for whom data on gender was available.

^3^
Descriptives based on *N* = 391 participants for whom data on age was available.

^4^
This timeframe was chosen as it (a) covers the period when most or all studies relevant to this synthesis have been published thus far, and (b) is relatively recent such that individual‐level data are more likely to be accessible; in addition (c) we collated and verified the data in July 2021.

^5^
Two additional studies (Kessler et al., 2020; Krans et al., 2010) fulfilled the first two criteria, but it was not possible to solve data‐sharing concerns (due to, e.g., GDPR) in time before finalizing the preregistration for the current manuscript.

^6^
Note a discrepancy of nine participants between the final sample stated in the pre‐registration (*N* = 587) and in this manuscript (*N* = 578). This is because it became apparent during the analysis stage that for those nine participants either diary data or IES/IES‐R data was missing and thus no correlation between the two variables could be computed.

^7^
Except one study that assessed the IES‐R after 24 h (Woud, Blackwell, et al., 2018)
